# *Pelargonium graveolens* Aqueous Decoction: A New Water-Soluble Polysaccharide and Antioxidant-Rich Extract

**DOI:** 10.1155/2018/2691513

**Published:** 2018-11-12

**Authors:** Malek Ennaifer, Taroub Bouzaiene, Moncef Chouaibi, Moktar Hamdi

**Affiliations:** ^1^University of Carthage, Institut National des Sciences Appliquées et de Technologie (INSAT), 1080 Tunis, Tunisia_LETMI – INSAT, Tunisia; ^2^Higher Institute of Food Industry, Tunis (ESIAT), Tunisia

## Abstract

**Background:**

The decoction of* Pelargonium graveolens* yields an antioxidant-rich extract and a water-soluble polysaccharide. This study aims (1) to investigate the effect of process parameters (extraction time and temperature) on the antioxidant activity of the decoction and the extraction yield of CPGP by response methodology and (2) to study the chemical properties of the optimized decoction and rheological properties of the corresponding extracted polysaccharide.

**Results:**

The antioxidant-rich decoction contained about 19.76 ± 0.41 mg RE/g DM of flavonoids and 5.31 ± 0.56 mg CE/gDM of condensed tannins. The crude* Pelargonium graveolens* polysaccharide (CPGP) contained 87.27 % of sugar. Furthermore, the CPGP solutions (0.5%, 1%, and 2%) exhibited shear-thinning or pseudoplastic flow behavior. A central composite design (CDD) was applied to assess the effects of temperature and time on the antioxidant activity of the decoction, on the one hand, and on water-soluble polysaccharide yield, on the other. The decoction optimization of* Pelargonium graveolens* aimed to use less energy (93°C for 11 minutes) leading to the highest values of decoction phenolic content (33.01 ±0.49 mg GAE/gDM) and DPPH scavenging activity (136.10 ± 0.62 mg TXE/gDM) and the highest values of CPGP yield (6.97%).

**Conclusion:**

The obtained results suggest that the CPGP rheological characteristics are suitable for applications in many industries, especially food. The values of optimal conditions showed that* Pelargonium graveolens* decoction operation could have multiple uses, especially for consuming less energy.

## 1. Introduction

*Pelargonium graveolens* is a herb belonging to the Geraniaceae family and it has good aromatic properties. It is cultivated worldwide [1, 2], mainly for its essential oil fraction, which is extensively used in many industries. The essential oil of the fresh plant is widely used in perfume industry thanks to its desirable scent [3]. Besides, many studies on active molecules in essential oil and organic extracts of* Pelargonium graveolens* have shown good antioxidant activity and antimicrobial effect, especially against* B. cereus*,* B. subtilis,* and* S. aureus *[4–6]. However, because of the toxicity of essential oils and organic extracts, their application in food against spoilage pathogens is limited, and more interest in safety matters should be shown [7].

In Tunisia, rose-scented geranium is broadly used to produce a food flavoring hydrosol used in traditional pastries. Some studies have proven its good antioxidant activity and capacity to heal throat pains [8]. Nevertheless, after distillation, the used plant is considered as waste. The decoction uses the whole plant and exhibits the presence of many active compounds such as phenolics [9, 10]. Yet, to our knowledge, little is known about the phytochemical composition and biological activities of* Pelargonium graveolens *decoction even though it is an ancestral practice used for its extract digestibility and safety compared to essential oils [10]. Moreover, decoctions are still used and even optimized to improve their added-value products [9, 11]. Decoction optimization parameters include temperature extraction, time extraction [9], pH, and the ratio of water to raw material [11]. Optimization was performed using experimental designs to produce response surfaces that were also widely used to determine optimal conditions to extract polysaccharides from different sources [12–14] and different processes [15]. Although most of new water-soluble polysaccharide extraction operations start with a decoction [14, 16, 17], no special interest has been given to both optimal extraction parameters leading to an added-value decoction and a maximum yield of its water-soluble polysaccharide.

This study aims (1) to investigate the effect of process parameters (extraction time and temperature) on the antioxidant activity of the decoction and the extraction yield of CPGP by response methodology and (2) to study the chemical properties of the optimized decoction and rheological properties of corresponding extracted polysaccharide.

## 2. Material and Methods

### 2.1. Plant Material and Sampling

Geranium aerial parts were harvested from a random sample of a plant growing in Ariana (North of Tunisia: latitude 36°51′36^″^ N, longitude 10°11′36^″^ E, altitude 10 m) in April 2014. Leaves, flowers, and stems were manually isolated from the branches to obtain a weight of 1.00 kg and dried at 20°C for 2 weeks. A specimen was kept in our unit as a reference.

### 2.2. Chemicals

All chemicals were purchased from Sigma, Tunisia.

### 2.3. Decoction Operation

**Figure 1:**
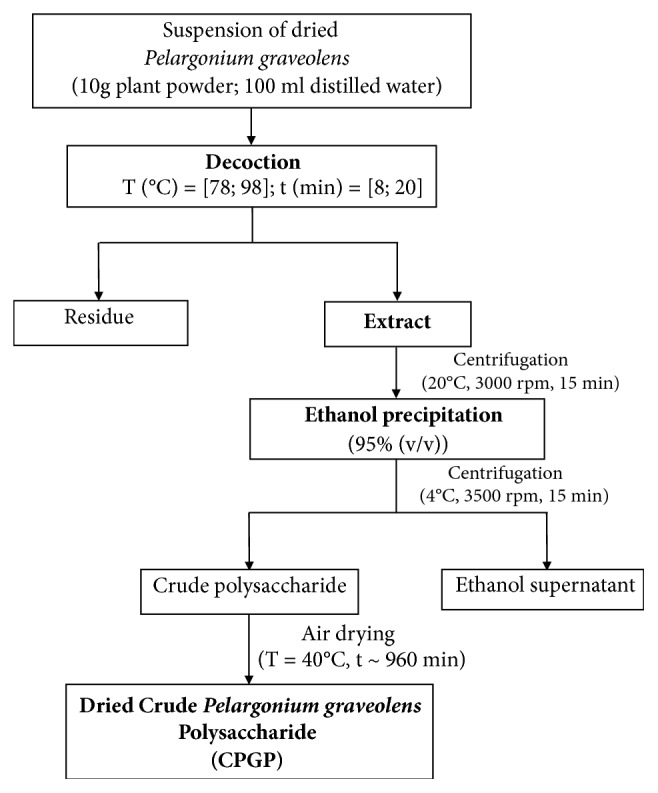
Process of* Pelargonium graveolens* decoction and polysaccharide extraction.

Dried ground* Pelargonium graveolens* whole plant (stems, flower, and leaves) (10 g) was extracted with distilled water (ratio of water to raw material (ml/g) was 10:1), while the water temperature was maintained at a given temperature (within ± 2°C, extraction temperature ranging from 78 to 98°C) for a given time (extraction time ranging from 8 to 20 min) (Figure 1). The flask was then cooled and the mixture was then filtered over a Buchner funnel. The decoction was prepared in triplicate. The resulting decoctions were stored at 4°C for future use.

### 2.4. Crude Polysaccharide Extraction

After decoction centrifugation, a volume of ethanol was added to the supernatant. The mixture was shaken overnight at room temperature (400 mo./min). Then, the solution was centrifuged for a quarter of an hour (4°C; 3500 rpm); the resulting precipitate was collected; the crude* Pelargonium graveolens* polysaccharide (termed CPGP) was obtained. The extract was air dried at 40°C until constant weight (Figure 1). The CPGP yield (%) was calculated by the equation [18]:

(1) $$\begin{array}{c} CPGP\;\;yield\left(\;\%\;\right)=\frac{m_{0}}{m}\;\;x\;\;100 \end{array}$$

where m_0_ (g) is the dried CPGP weight and m (g) is the dried raw material (DM) weight.

### 2.5. Optimization of Decoction

The central composite design (CDD) was applied to study the effect of temperature (X1) and time (X2) on the total phenolic content (Y1), DPPH essay (Y2), and yield of CPGP (Y3) as responses. The experimental factors and levels are shown in Table 1. The coded levels and experimental value of each factor, in each experience, are shown in Table 2.

The polynomial model used to express the responses was

(2) Y=b0+b1∗X1+b2∗X2+b11∗X1∗X1+b22∗X2∗X2+b12∗X1∗X2

where Xi represents the level of the factor i, Y is the experimental response, and b is a parameter of the model (regression coefficient).

Every model parameter has a precise meaning: b0 represents the response analyzed at the domain center; the values of b1 and b2 indicate the importance of the effects of the factors (temperature and time, respectively) on the responses; b12 is an interaction parameter between the two factors. The values of b11 and b22 determine the movement of the response surface (upward for positive values or downward for negative values) [9].

### 2.6. Physical Chemistry of the Decoction

#### 2.6.1. Total Phenolic Content

The Folin–Ciocalteu method [19] was used to assess the total phenolic content. The phenol contents were expressed as milligrams of Gallic acid equivalent per gram of dry matter (mg GAE/gDM).

#### 2.6.2. Flavonoid Contents

The colorimetric method [20] was used to measure the flavonoid contents. Briefly, 0.5 mL of each diluted extract was mixed with 0.5 mL of 2% AlCl_3_ methanol solution. After 30 min incubation, the absorbance was read at 430 nm. Flavonoid contents were calculated from a calibration curve of rutin and expressed as milligrams of rutin equivalent per gram of dry matter (mg RE/gDM). The results are means of triplicates.

#### 2.6.3. Total Condensed Tannins

To measure the condensed tannins, the vanillin assay [21] was performed. To 50 *μ*l of diluted sample, a volume of methanol vanillin solution (3 ml, 4%) and a volume of H_2_SO_4_ (1.5 ml) were added. After a 15 min reaction, the absorbance was read at 500 nm. The methanol was used as a blank. The amount of total condensed tannins was expressed as milligrams of catechin equivalent per gram of dry matter (mg CE/gDM). All samples were analyzed in three replications.

#### 2.6.4. Free Radical Scavenging Activity

The DPPH (2,2- diphenyl-1-picrylhydrazyl) radical scavenging capacity was measured [22]. Briefly, 50*μ*L of a double serial dilution of the aqueous extracts were mixed to 0.95 mL of 60 *μ*M DPPH radical solution and left away from light for 30 min. The spectrophotometer was set at 517 nm. The percentage of radical inhibition (I %) was estimated as

(3) $$\begin{array}{c} I\%=100\times \frac{\left(A_{0}-A_{1}\right)}{A_{0}} \end{array}$$

where A_0_ is the control absorbance and A_1_ is the sample absorbance. All tests were triplicated. For the optimized decoction extract, the DPPH activity was calculated from a calibration curve of Trolox and expressed as milligrams of Trolox equivalent per gram of dry matter (mg TXE/gDM).

#### 2.6.5. Color Measurement

The CIELAB coordinates (L*∗*, a*∗*, b*∗*) were measured in a Minolta colorimeter (Minolta, Model CM-3600 d, UK) controlled by a computer that calculated color from the reflectance spectrum [23]. The L*∗* parameter (lightness index) ranges from 0 (black) to 100 (white). However, the a*∗* parameter indicates the degree of red (+a*∗*) or green (-a*∗*) colors, whereas the b*∗* parameter measures the degree of the yellow (+b*∗*) or blue (-b*∗*) colors. Samples were poured in Petri dishes till the brim and placed on the device sensor.

### 2.7. Sugar Content, FTIR Spectra, and Rheology of CPGP Solutions

The* Pelargonium graveolens* decoction was first treated with the Sevag reagent to eliminate any resulting proteins [24]. Next, the supernatant was dialyzed for three days and, finally, the CPGP was precipitated using ethanol (V/V). The obtained crude polysaccharide was dried at 40°C until constant weight, then suspended in distilled water to measure the sugar content [18]. For the FTIR test, the CPGP was rather lyophilized than dried before spectroscopy experiment.

#### 2.7.1. Sugar Content

The phenol-sulphuric method was used [25]. The purity (%) of CPGP is calculated as the sugar content of extraction per dried crude polysaccharide weight.

#### 2.7.2. Fourier Transform Infrared Spectroscopy

The Fourier Transform Infrared (FTIR) method was used to characterize CPPG by a VERTEX 70 (Bruker Optics, USA) spectrometer. The decoction was further deproteinized by the Sevag reagent (a mixture of CHCl_3_ and n-butanol, v/v = 4:1). The aqueous fraction was precipitated by adding ethanol. The mixture was centrifuged and the crude polysaccharide (the precipitate) was then suspended in water and dialyzed for three days. The precipitate was lyophilized. The FTIR spectral bonds ranged from 500 to 4000 cm^−1^.

#### 2.7.3. Viscosity Measurement

The flow behavior of different CPGP water solutions (0.5; 1 and 2%) was measured by a strain-controlled rheometer (AR 2000, TA Instruments, Ltd., Crawley, UK). The viscosity was measured at a temperature of 20°C and shear rates between 10 s^−1^ and 1000 s^−1^. Flow behavior was determined by the power law model:

(4) $$\begin{array}{c} \sigma =k\gamma^{n} \end{array}$$

where *σ* is the shear stress (Pa), *γ* is the shear rate (1/s), n is the flow index, and k is the consistency index.

### 2.8. Statistical Analysis

The statistical analysis was performed using one way analysis of variance (ANOVA) followed by Duncan's test for the means comparisons and a* p* value of less than 0.05 was considered significant.

## 3. Results

### 3.1. Optimization of the Decoction of* Pelargonium graveolens*

There were 16 runs for optimizing the five individual parameters in the current CDD design (Table 2). The data were analyzed by multiple regression analysis using NEMRODW (9901- by LPRAI_Marseille, France) and each response was studied separately.

#### 3.1.1. Model Validation

The statistical analysis checks the existence of coefficients which do not influence responses. A good and correct description of the model variation of the test results can be affirmed when the significance shown in the tables of the analysis of variance is superior to 95% [26–28].

Table 3 lists the obtained results of the statistical test, the estimated values of the model coefficients, and the model validation parameters. For response Y1, only 2 parameters were significant in decoction process. Thus, total phenolic content of the decoction could be given by the equation:

(5) $$\begin{array}{c} Y1=32.524+3.191\;\;X1 \end{array}$$

According to this equation, the temperature may have a linear effect on the total phenolic content. For response Y2, 2 out of the 6 model parameters were significant in decoction process. Similarly to the case of the response Y1, the coefficients b2-b12 do not influence the response since these coefficients' value of significance is less than 95%. Thus, the decoction antioxidant activity could be given by the equation:

(6) $$\begin{array}{c} Y2=77.72+8.227\;\;X1 \end{array}$$

According to this equation, the temperature may have a linear effect on the DPPH scavenging activity. The statistical analysis for response Y3 shows that only three parameters were significant in decoction process. Thus, polysaccharide yield extract from the decoction could be given by the equation:

(7) $$\begin{array}{c} Y3=4.937+1.702\;\;X1+0.738\left(\;X2.X2\;\right) \end{array}$$

According to this equation, the temperature may have a linear effect on CPGP yield. However, the extraction time may have a quadratic effect on the same response.

#### 3.1.2. Extraction Parameters Influence on Decoction Total Phenolic Content

**Figure 2:**
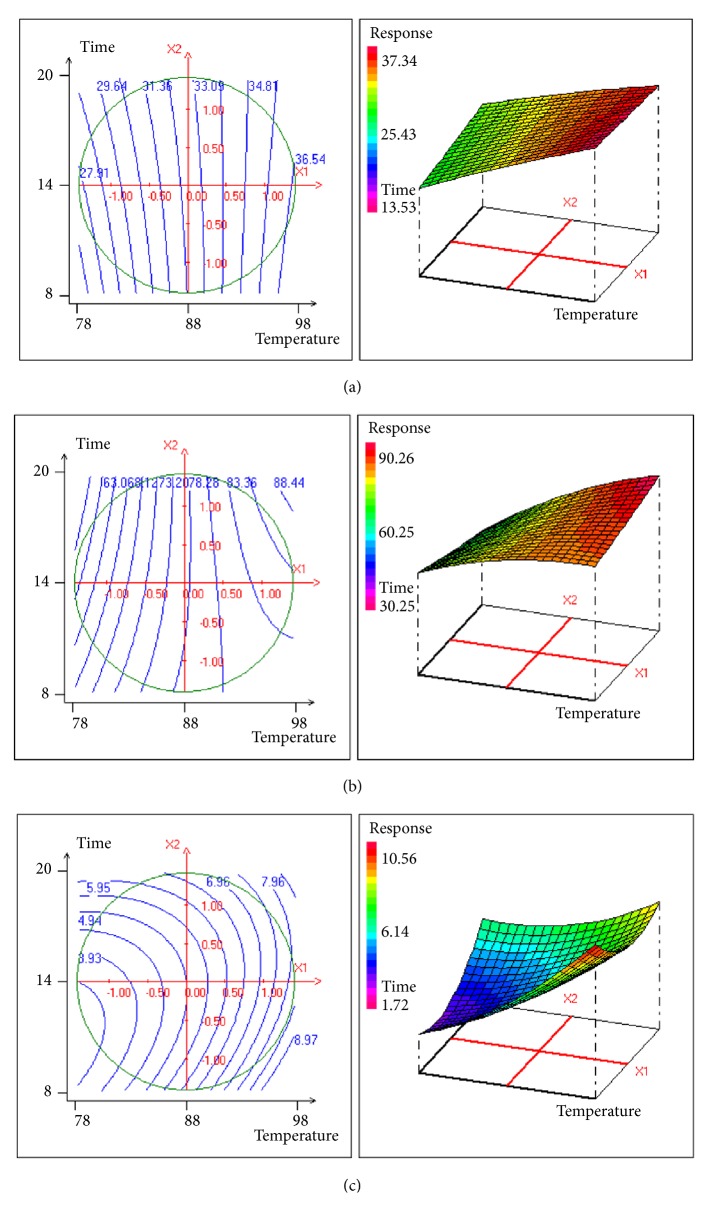
Contour plots and 3D-response surfaces for (a) total phenolic content, (b) DPPH scavenging activity, and (c) CPGP yield, as a function of time and temperature of decoction.

According to the positive linear coefficient of (5) (+3.191), the phenolic content reaches higher values with increase in extraction temperature. This can be observed in Figure 2(a), showing the contour plots for phenolic content. The area of the experimental domain shows that, independent of time, the amount of total phenolics reaches its maximum for a temperature interval between 88°C (X1=0.5) and 98°C (X1=+ *α*). Within this interval, about 37% of phenolic content is obtained (Figure 2(a)_3D surface plot).

#### 3.1.3. Extraction Parameters Influence on Decoction Scavenging Activity

As time is kept constant, a difference in temperature increases the scavenging activity.  Figure 2(b) shows that the scavenging activity reaches its maximum for a temperature extraction interval between 88°C (X2=0.5) and 98°C (X2=+ *α*). The time of extraction would have an impact only for extreme temperature.

#### 3.1.4. Extraction Parameters Influence on Crude* Pelargonium graveolens* Polysaccharide Yield

According to (7), the time and temperature of the extraction increase the CPGP yield.  Figure 2(c) shows that the yield of polysaccharide CPGP reaches its maximum at an interval of temperature between 95°C (+1) and 98°C (+ *α*), regardless of decoction time.

#### 3.1.5. Predicted and Experimental Optimal Condition of Total Phenolic and CPGP Contents

The main objective of this study is to look for the optimum parameters values which help to enhance the decoction antioxidant activity and the corresponding polysaccharide yield. Tables 4 and 5 present optimal conditions and predicted variables. In an experiment with X1 of about 0.773 (temperature of the order of 93°C) and X2 of -0.634 (time of 11 minutes), the desirability for phenolic content (Y1 close to 34.98 mg GAE/gDM), scavenging activity (Y2 close to 82.10%), and the yield of water-soluble polysaccharide (Y3 close to 6.97%) is maximal (98.84%). Hence, the values of the parameters mentioned here before strengthen the probability of having an optimum.

To ensure the predicted result, test rechecking was conducted using the modified optimal conditions of temperature extraction of 94°C and decoction time of 10 minutes.  Table 6 showed that the experimental results did not vary a lot from the predicted value.

### 3.2. Physical Chemistry of Optimal Decoction

The aqueous extract represented 51.41% of the starting mixture (into-water dry material) which was nearly six times the weight of the used dry material (DM). The decoction had an acid pH, about 4.32. It had a liquid appearance (viscosity about 1.33 Pa.s) and a color similar to dark tea infusion, whose characteristics are presented in Table 7.

The total phenolic content, flavonoids, and condensed tannins of the optimal* Pelargonium graveolens *decoction were measured to reflect their biological property as expressed in DPPH scavenging activity (Table 8).

### 3.3. *Pelargonium graveolens* Crude Polysaccharide (CPGP) from Optimal Decoction

In this work, the rheological properties of the CPGP and its preliminary structural analysis were studied.

#### 3.3.1. Rheological Properties of Crude* Pelargonium graveolens* Polysaccharide

**Figure 3:**
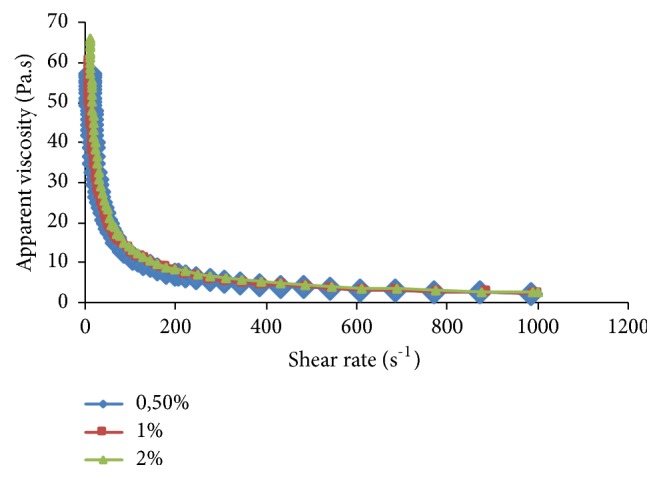
Flow behaviors of crude* Pelargonium graveolens* polysaccharide at different concentrations (0.5, 1, and 2 %; w/v).

The rheology of* Pelargonium graveolens* water solutions was studied using the behavior of the apparent viscosity versus the shear rate (Figure 3). The graph showed two areas: the apparent viscosity decreased until a shear rate of 400 s^−1^, then a constant *«*unlimited*»* viscosity was established. This rheological property characterizes the pseudoplastic or shear-thinning behavior (n<1).

**Figure 4:**
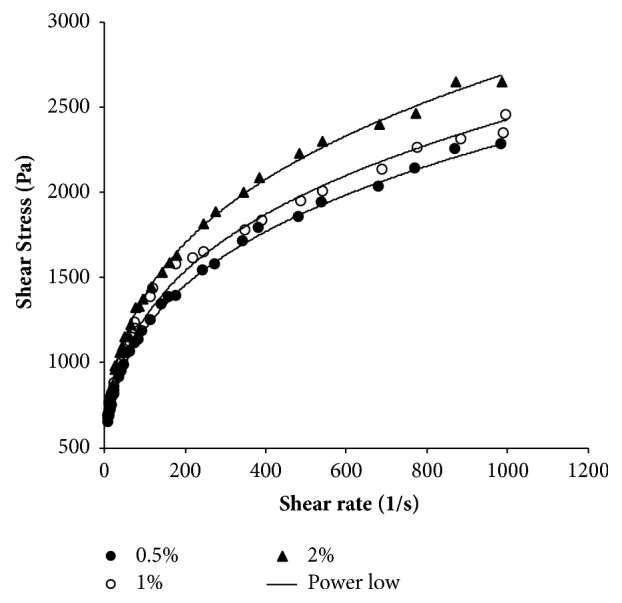
Shear stress versus shear rate of crude* Pelargoinum graveolens* polysaccharide water solution at different concentrations.

The flow behavior index (n) and consistency index (k) values (Table 9) were obtained from the representation of the shear stress versus shear rate according to the power law model (Equation (4), Figure 4). The results showed that the increase in concentration increases the shear stress. Table 9 shows that the flow index decreases with the increase in the concentration of polysaccharide. The statistical analysis revealed that there are no significant differences between all concentrations in terms of index flow.

However, the consistency index values ranged from 322.60 to 382.70 Pa·s^n^. There were significant variations of index consistency of all samples.

#### 3.3.2. Preliminary Structural Analysis

For preliminary CPGP structural analysis purpose, the sugar content by the phenol-sulphuric method and the Fourier Transform Infrared spectra were measured.

**Figure 5:**
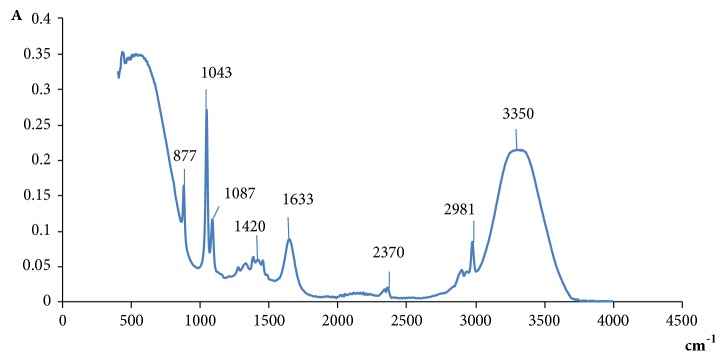
Fourier transform infrared spectra of extracted CPGP.

The total sugar content of CPGP was estimated to be 87.27% and Figure 5 shows the FTIR spectra of CPGP, exhibiting a large absorption band at around 3350 cm^−1^, four weak peaks at 2981 cm^−1^, 2370 cm^−1^, 1633 cm^−1^ and 1410 cm^−1^. Two intense absorption bands were also observed at 1043 cm^−1^ and 1087 cm^−1^. Finally, an absorption band at around 877 cm^−1^ marks the beginning of the *«*fingerprint*»* area.

## 4. Discussion

The central composite design was applied to assess the effect of temperature and time on the phenolic content and the antioxidant activity of the decoction. The response surface analysis revealed that the temperature was the most impacting factor. Indeed, the response reached the maximum value with the increase in temperature. This suggests that the temperature has an effect on plant tissue in improving the phenolic compound extraction [9]. Such finding was recurrent in many studies, stating that the increase in extraction time and temperature enhances the material particles solubility [29] and the diffusion coefficient [30].

Besides, maintaining a relatively high temperature would increase the yield of the crude* Pelargonium graveolens* polysaccharide (CPGP). Indeed, different herbal water-soluble polysaccharides extraction studies showed that the extraction temperature varied from 48,7°C to 100°C [4, 12–14, 31–34]. However, to enhance the macromolecules yield, the time of extraction varied from 29 minutes to 4 hours [15, 33]. At these extraction conditions, the polysaccharide yield varied from 5,37% to 18,88 % [12–14, 32–34]. But, since the yield of polysaccharide was the unique matter of these studies, no attention was paid to the activity of other decoction molecules at prolonged heat treatment.

After the validation of optimal extraction conditions, the* Pelargonium graveolens* decoction at 94°C for 10 minutes presented a particular brown color. For tea, this color is desirable [35] and it is influenced by the polyphenol oxidase activity which oxidizes polyphenols to flavonoids, catechins, and brown colored compounds [35, 36]. For tea infusion, the L*∗* value is about 59.03; however, for fermented tea infusion it is about 40.25, whereas the lightness of the decoction is much lower (18.74), which may correspond to higher dark colored compounds like those developed through tea fermentation [36]. The redness (a*∗*) and yellowness (b*∗*) of tea infusions (26.17-59.03 and 15.17-25.71, respectively) are, however, higher than color parameters of the optimal decoction (2.17 and 11.07). These parameters could help to differentiate between various* geranium* species extracts using different processes [36] (infusion, decoction, cold extraction...).

The value of the radical scavenging activity of the optimal* Pelargonium graveolens *decoction was about 136.1 mgTXE/gDM (68.05%). This finding was explained by the presence of phenolic components (flavonoids and condensed tannins) and mostly flavonoids (19.77 mgRE/gDM) [10, 37]. In addition, the studies of the antioxidant and phenolic profiles of* Pelargonium graveolens* hydrosols, aqueous extracts, methanol extracts, and essential oils exhibited phenolic contents varying from 54.71 mgGAE/gDM to 102.44 mgGAE/gDM and scavenging activity up to 83% [2, 38, 39].

Roseiro et al. (2013) [9] reported that, under optimum extraction temperature and time (98.5°C and 17 min, respectively), carob kibbles decoction exhibited a DPPH scavenging activity of about 85% and total phenolic content of about 39.5 mg GAE/gDM.

The* Pelargonium graveolens *water solutions exhibited a pseudoplastic or shear-thinning behavior. Adeli and Savmati (2014) [33] reported that the flow behavior index (n) of 1.5 % w/v Ziziphus lotus fruit polysaccharide solution (WPZL) was about 0.77, while the same index for the CPGP at 1% was about 0.27. This can be explained by the fact that the viscosity of CPGP is higher than that of WZPL at 1% w/v concentration.

The novel water-soluble polysaccharide had a total sugar content of 1.42 time higher than boat-fruited sterculia seeds (61.17%) [40] and 1.19 times higher than chickpea polysaccharide [41].

An attempt to a structural analysis of the CPGP by the Fourier Transform Infrared has revealed the presence of a large absorption band at around 3350 cm^−1^, which may be associated with a hydroxyl group [14]. In fact, as reported by Chien et al. (2015) [42], the peaks from 3200 to 3600 cm^−1^ may be associated with O-H groups. Furthermore, the band detected at 2981 cm^−1^ indicated the stretching vibration of C-H groups [34, 42]. The presence of an absorption band at 1633 cm^−1^ suggests the presence of carboxylate stretching group (COO^−^) [14] for a band peak around 1605 cm^−1^. Nevertheless, other studies associated the absorption bands from 1640 cm^−1^ to 1651 cm^−1^ with C=O groups [42]. However, at 1642 cm^−1^, the band was associated with water [31]. Moreover, the peak at 1420 cm^−1^ was assigned to C-O stretching vibration [31] and suggests the presence of uronic acid content [13, 43]. A strong absorption band was also observed at 1043 cm^−1^ that could be associated with the C-O-C stretching vibration of glycosidic structure [14] and might suggest the presence of pyranose ring (1043 to 1087 cm^−1^) [31] or even furanose [34, 44].

The obtained bands at 890 cm^−1^ suggest the presence of the *β*-glycosidic bond [42] or *β*-D-glucan [45]. It was also reported that the absorption bands between 810 and 870 cm^−1^ could suggest the presence of mannan in the studied sample [42, 46].

## 5. Conclusion

The response surface methodology was used in this study to improve the antioxidant potential of the decoction of* Pelargonium graveolens* and to enhance the yield of the polysaccharide extraction. The extraction temperature had a linear effect on the different responses and the extraction time had a quadratic effect only on the polysaccharide yield. Besides, there was no interaction between the two extraction parameters. The optimal extraction conditions were obtained: extraction temperature (93°C) and extraction time (11 min). Under these conditions, different process responses were as follows: the phenolic content was 33.02%, the radical scavenging activity was 68.05%, and the CPGP yield was 6.43%. These results are in good agreement with the predicted values.

The crude* Pelargonium graveolens* polysaccharide solutions were found to exhibit shear-thinning non-Newtonian flow behavior for concentrations above 0.5% (w/v). The obtained results suggest that the CPGP rheological characteristics are suitable for applications in many industries, especially food. Moreover, the values of optimal conditions showed that decoction operation could have multiple uses, especially for consuming less energy.

## Figures and Tables

**Table 1:** Experimental factors and their levels in CCD.

		Surface				
		Levels			Star points^*∗*^	
Factor	Unit	-1	0	1	- *α*	+*α*

(X1) Extraction temperature	°C	81	88	95	78	98

(X2) Extraction time	min	10	14	18	8	20

^*∗*^ *α* = 1.41.

**Table 2:** Central composite design for the decoction: temperature and time, with observed responses (total phenolic content, DPPH scavenging activity, and crude *Pelargonium graveolens *polysaccharide yield).

TEST	*Factors*				*Responses*		
	**X1**		**X2**		**Y1**	**Y2**	**Y3**

	Temperature (°C)	Coded level	Time (min)	Coded level	Total phenolic content (mg GAE/gDM)	DPPH scavenging activity (%)	CPGP yield (%)

1	81	-1	10	-1	28.24	72.9	3.48

2	95	1	10	-1	36.04	81.93	8.97

3	81	-1	18	1	30.37	65.13	4.11

4	95	1	18	1	36.36	85.92	7.78

5	88	0	14	0	30.79	68.7	7.25

6	88	0	14	0	33.8	77.2	4.99

7	78	-1.41	14	0	27.05	60.5	4.06

8	98	1.41	14	0	36.54	88.44	8.21

9	88	0	8	-1.41	32.27	79.41	5.64

10	88	0	20	1.41	32.4	76.47	7.33

11	88	0	12	-0.5	31.64	73.11	4.68

12	88	0	16	0.5	31.63	83.19	5.48

13	84	-0.5	14	0	26.86	67.65	3.69

14	92	0.5	14	0	34.42	81.3	5.95

15	79	-1.3	12	-0.5	28.47	65.69	3.43

16	97	1.3	16	0.5	35.54	83.82	6.93

**Table 3:** Parameters of the polynomial models representing the studied responses (Y1-Y3).

***Model***	Y1		Y2		Y3	
***Model parameters***	Coefficient	*P* value	Coefficient	*P* value	Coefficient	*P* value

b0	32.524	*∗∗∗*	77.720	*∗∗∗*	4.937	*∗∗∗*

b1	3.911	*∗∗∗*	8.227	*∗∗∗*	1.702	*∗∗∗*

b2	0.224	n.s	-0.503	n.s	0.195	n.s

b11	-0.161	n.s	-1.976	n.s	0.446	n.s

b22	0.032	n.s	0.277	n.s	0.738	*∗*

b12	-0.442	n.s	2.508	n.s	-0.562	n.s

***Model validation***						

Significance level (%)	*∗∗∗*		*∗∗∗*		*∗∗∗*	

Df	13		13		13	

Sum of squares	1.28E+02		9.16E+02		42.341	

Mean square	24.04		1.66E+02		7.673	

**R** ^**2**^	0.949		0.910		0.902	

**Adjusted R** ^**2**^	0.918		0.854		0.847	

*∗∗∗*: Significant at the level 99.9%

*∗∗*: Significant at the level 99%

*∗*: Significant at the level 95%

n.s: not significant

Df: degrees of freedom.

**Table 4:** Optimal conditions for the extraction process.

Variable	Value	Factor	Value
X1	0.773326	Temperature	93

X2	-0.634008	Time	11

**Table 5:** Predicted values of the responses at optimal conditions.

**Response**	**Name**	**Value **	**di **%	**Weight**	**di min **%	**di max **%
Y1	Total phenolic content **(mgGAE/gDM)**	**34.98**	99.65	1	42.51	99.65

Y2	DPPH scavenging activity **(**%**)**	**82.10**	98.43	1	48.69	98.43

Y3	CPGP yield **(**%**)**	**6.97**	98.45	1	63.20	98.45

	**DESIRABILITY**		98.84		50.76	98.84

di: percentage of calculated desirability.

**Table 6:** Predicted and experimental values of responses at optimal and modified conditions.

	**Extraction temperature ** **(**°**C)**	**Extraction time ** **(min)**	**Total phenolic content ** **(mg GAE/gDM)**	**DPPH scavenging activity ** **(**%**)**	**CPGP yield ** **(**%**)**
**Predicted values**	93	11	34.98	82.10	6.97

**Modified conditions**	94±2	10	33.02 ±0.58	68.05±0.74	6.43±0.31

**Table 7:** Color (L, a, b) index of the *Pelargonium graveolens* optimized extract.

**Sample**	**L** **∗**	**a** **∗**	**b** **∗**
Decoction extract	18,74 ± 0,68	2,17 ± 0,33	11,07 ± 0,63

L*∗*: Lightness, a*∗*(-green/+red), b*∗*(-blue/+yellow).

**Table 8:** Chemical content and antioxidant properties of *Pelargonium graveolens* optimized decoction extract.

**Sample**	**Total phenolic content ** **(mgGAE/gDM)**	**Flavonoids ** **(mg RE/gDM)**	**Condensed tannins ** **(mg CE/gDM)**	**DPPH scavenging activity ** **(mg TXE/gDM)**
**Optimal decoction extract**	33.01 ± 0.49	19.76 ± 0.41	5.31 ± 0.56	136.10 ± 0.62

**Table 9:** Flow behavior index (n) and consistency index (k) of CPGP at different concentrations.

**Concentration (w/v) (**%**)**	**n**	**K (Pa·s** ^**n**^ **)**	**R** ^**2**^
0,5	0.283±0.01^a^	322.60±5.80^a^	0.998

1	0.277±0.02^b^	354.60±7.50^b^	0.995

2	0.279±0.01^c^	382.70±6.94^c^	0.999

The different letters indicated significant difference at p<5%.

## Data Availability

The data used to support the findings of this study are available from the corresponding author upon request.
